# Perinatal outcomes of singleton live births after late moderate-to-severe ovarian hyperstimulation syndrome: A propensity score-matched study

**DOI:** 10.3389/fendo.2022.1063066

**Published:** 2022-12-01

**Authors:** Shiyu Ran, Ruowen Zu, Huan Wu, Wei Zheng, Chen Yang, Shuheng Yang, Bingnan Ren, Wen Zhang, Jiangbo Du, Yichun Guan

**Affiliations:** ^1^ Department of Reproductive Medicine Center, The Third Affiliated Hospital of Zhengzhou University, Zhengzhou, Henan, China; ^2^ State Key Laboratory of Reproductive Medicine (Henan Centre), The Third Affiliated Hospital of Zhengzhou University, Zhengzhou, Henan, China; ^3^ State Key Laboratory of Reproductive Medicine, Nanjing Medical University, Nanjing, Jiangsu, China; ^4^ Department of Epidemiology, Center for Global Health, School of Public Health, Nanjing Medical University, Nanjing, Jiangsu, China

**Keywords:** ovarian hyperstimulation syndrome, *in vitro* fertilization, intracytoplasmic sperm injection, perinatal outcomes, normal term infant

## Abstract

**Objective:**

To evaluate whether singleton live births achieved following *in vitro* fertilization (IVF)/intracytoplasmic sperm injection (ICSI) in women with late moderate-to-severe ovarian hyperstimulation syndrome (OHSS) is associated with adverse perinatal outcomes.

**Methods:**

This was a single-center retrospective cohort study conducted from January 2016 to June 2021. A total of 4,012 IVF/ICSI-fresh embryo transfer cycles that achieved singleton live births were included. According to the diagnosis of OHSS, the cycles were divided into two groups: late moderate-to-severe OHSS (MS-OHSS) group (*n* = 114) and non-OHSS group (*n* = 3,898). Multiple baseline covariates were controlled by propensity score matching, yielding 114 late MS-OHSS singleton live births matched to 337 non-OHSS singleton live births. The primary outcome of the study was normal term infant. The secondary outcomes were perinatal complications, gestational age at birth, birth weight, and birth height.

**Result(s):**

Before propensity score matching, no significant difference in perinatal outcomes was identified between late MS-OHSS group and non-OHSS group. After matching maternal age, BMI, basal serum FSH level, basal serum AMH level, basal antral follicle count, type of stimulation protocol, day of embryo development for embryo transfer, number of embryo transfer, and number of oocytes retrieved, there was still no significant difference in obstetric outcomes and neonatal outcomes between the two groups.

**Conclusion(s):**

The findings demonstrate that the perinatal outcomes were similar between the two groups. However, because the sample size of patients with late MS-OHSS was limited in this study, further investigations are warranted using a larger sample size.

## Introduction

Ovarian hyperstimulation syndrome (OHSS) is an iatrogenic complication of assisted reproductive technology (ART) and can occur in any woman who undergoes ovulation induction (1). The incidence of moderate-to-severe (MS) OHSS in women undergoing ART is 3% to 8% (2). MS-OHSS is characterized by enlarged ovaries, high concentrations of sex steroid hormones and the accumulation of extravascular exudate (2, 3). Additionally, abdominal bloating, abdominal pain, ascites, hydrothorax, oliguria and anuria are common clinical manifestations (3). Potential complications, such as renal failure and oliguria, hypovolemic shock, thromboembolic episodes, adult respiratory distress syndrome, and even death, mean MS-OHSS is potentially life-threatening (2, 4, 5).

OHSS not only increases the financial burden of patients but also causes them physical and mental suffering (6). Mild OHSS does not need any treatment except for rest and routine follow-up. Patients with MS-OHSS are generally admitted to hospital and treated with fluid resuscitation, supportive care, paracentesis and prophylactic anticoagulation (7). However, the best course of action in women undergoing ART is to prevent the development of OHSS, which will requires a greater understanding of the pathophysiology of OHSS. It is believed that the key factor in the pathogenesis of OHSS is human chorionic gonadotrophin (HCG), which induces the secretion of vasoactive substances (8–10), such as vascular endothelial growth factor, leading to increased systemic vascular permeability (11, 12) and consequent massive transudation of protein-rich fluid from the vascular space into the peritoneal cavity and, to a lesser extent, the pleural and pericardial cavities (13).

Numerous studies on the prevention of OHSS have been published. However, few studies have shown whether pregnancies conceived after OHSS are associated with an increased risk of adverse perinatal outcomes. Three recent studies have reported that perinatal outcomes are comparable between women with and without OHSS (14–16). Nevertheless, several studies have found that OHSS may be correlated with an increased risk of preterm birth or low birth weight (LBW) (17–21). Recently, Hu et al. found that the incidence rates of thrombosis, gestational diabetes mellitus (GDM) and neonatal intensive care unit (NICU) admission were significantly higher in patients with MS-OHSS than in those without OHSS (22). However, these conclusions were based on limited data, and some studies have not ruled out multiple pregnancies and other factors that could be responsible. Therefore, the effects of late MS-OHSS on the perinatal outcomes of singleton live births warrant further investigation.

The aim of this retrospective cohort study was to investigate the effects of late MS-OHSS on obstetric and neonatal outcomes of single live births conceived following *in vitro* fertilization (IVF)-/intracytoplasmic sperm injection (ICSI)-fresh embryo transfer (ET) cycles.

## Materials and methods

### Study design and patients

In this study, we reviewed the medical records of all patients who underwent IVF-/ICSI-fresh ET cycles in the Center of Reproductive Medicine, the Third Affiliated Hospital of Zhengzhou University, from January 1, 2016, to June 30, 2021. All of the patients had undergone controlled ovarian hyperstimulation. Only singleton live births conceived after IVF-/ICSI-fresh ET cycles were included. The diagnosis and severity grading of OHSS were based on the criteria reported by Golan et al. (6). The classification of OHSS symptoms were as follows: mild OHSS: abdominal distension and discomfort, nausea, vomiting, enlarged ovaries, diarrhea; moderate OHSS: mild features, ultrasonic evidence of ascites; severe OHSS: moderate features, clinical evidence of ascites, hydrothorax or breathing difficulties, change in blood volume, increased blood viscosity due to hemoconcentration, coagulation abnormalities, diminished renal perfusion and function. The exclusion criteria were as follows: involved donated oocytes or donated sperm, mild OHSS, and patients with abnormal uterine anatomy, multiple pregnancies, recurrent spontaneous abortion, or recurrent implantation failure. Patients with moderate-to-severe OHSS were included in the late MS-OHSS group, and patients without OHSS were included in the non-OHSS group.

### Clinical protocols

All patients involved in this study were subjected a long protocol of gonadotropin -releasing hormone (GnRH)-agonists or GnRH-antagonists. The ovulation induction drug was recombinant and/or urinary gonadotrophins (Gonal-F, Merck, Serono, Germany) with or without luteinizing hormone (Luveris, Merck Serono, Germany). Experienced clinicians individually adjusted the starting dose of medication according to the patient’s age, body mass index (BMI), medical history and ovarian reserve. Recombinant human chorionic gonadotropin (r-HCG, Ovidrel, Merck Serono, Germany) at a dose of 5000 to 10000 IU was administrated after the dominant follicle reached 20 mm or at least three follicles reached 18 mm. Oocytes were retrieved under the transvaginal ultrasound monitoring 36-38 hours later (23). Then routine IVF or ICSI and embryo culture were performed (24). One or 2 cleavage-stage embryos were transferred on the third day or one blastocyst was transferred on the fifth day after fertilization. Oral dydrogesterone (Abbott Co. America) and progesterone sustained-release vaginal gel (Merck Co. Germany) were provided for luteal support. Beta-HCG serum concentrations were measured at 14 days after embryo transfer to determine whether biochemical pregnancy had been achieved (25). Clinical pregnancy was defined as the presence of an intrauterine gestational sac on transvaginal ultrasound at 4 weeks after transfer.

### Perinatal outcomes and their definitions

We obtained all patients’ characteristic data and IVF/ICSI treatment cycles data from the electronic medical record system of our hospital. The perinatal outcomes of pregnant patients were obtained through electronic medical record system and telephone follow-up. The primary outcome of this study was delivery of a normal term infant, defined as an infant born at the gestational age of ≥ 37 weeks to < 42 weeks (260 to 293 days), with weight > 2,500 g to < 4,000 g, and without any malformation or disease (26). The secondary outcomes were placenta previa, placental abruption (PA), premature rupture of membranes (PROM), hypertensive disorders of pregnancy (HDP), GDM, cesarean delivery, gestational age (weeks), preterm delivery (<37 weeks), birth weight (grams) [including macrosomia (>4,000 g), LBW (<2,500 g), very LBW (<1,500 g), small for gestational age (SGA), and large for gestational age (LGA) (27)], birth height (centimeters), admission to the pediatrics department, and birth defect (28, 29).

### Statistical analysis

SPSS 26.0 (IBM, Chicago, IL, USA) was used for statistical analyses. Continuous variables are presented as means ± standard deviations, and Student’s *t*-test was used to compare the differences between the two groups. Categorical data are presented as frequencies and corresponding percentages, and chi-square tests and Fisher’s exact tests were performed to assess the differences between the two groups. Logistic and linear regression analyses were used to assess perinatal outcomes. Odds ratios and adjusted odds ratios are reported with 95% confidence intervals. Two-tailed *P* values < 0.05 were considered statistically significant.

Maternal age, BMI, basal serum FSH level, basal serum AMH level, basal antral follicle count, type of stimulation protocol, day of embryo development for embryo transfer, number of embryo transfer, and number of oocytes retrieved were matched with propensity score matching (PSM) to control for confounding factors. To optimize the precision of the study, patients with late MS-OHSS were matched to patients without OHSS in a 1:3 ratio.

## Results

### Study population

**Figure 1:**
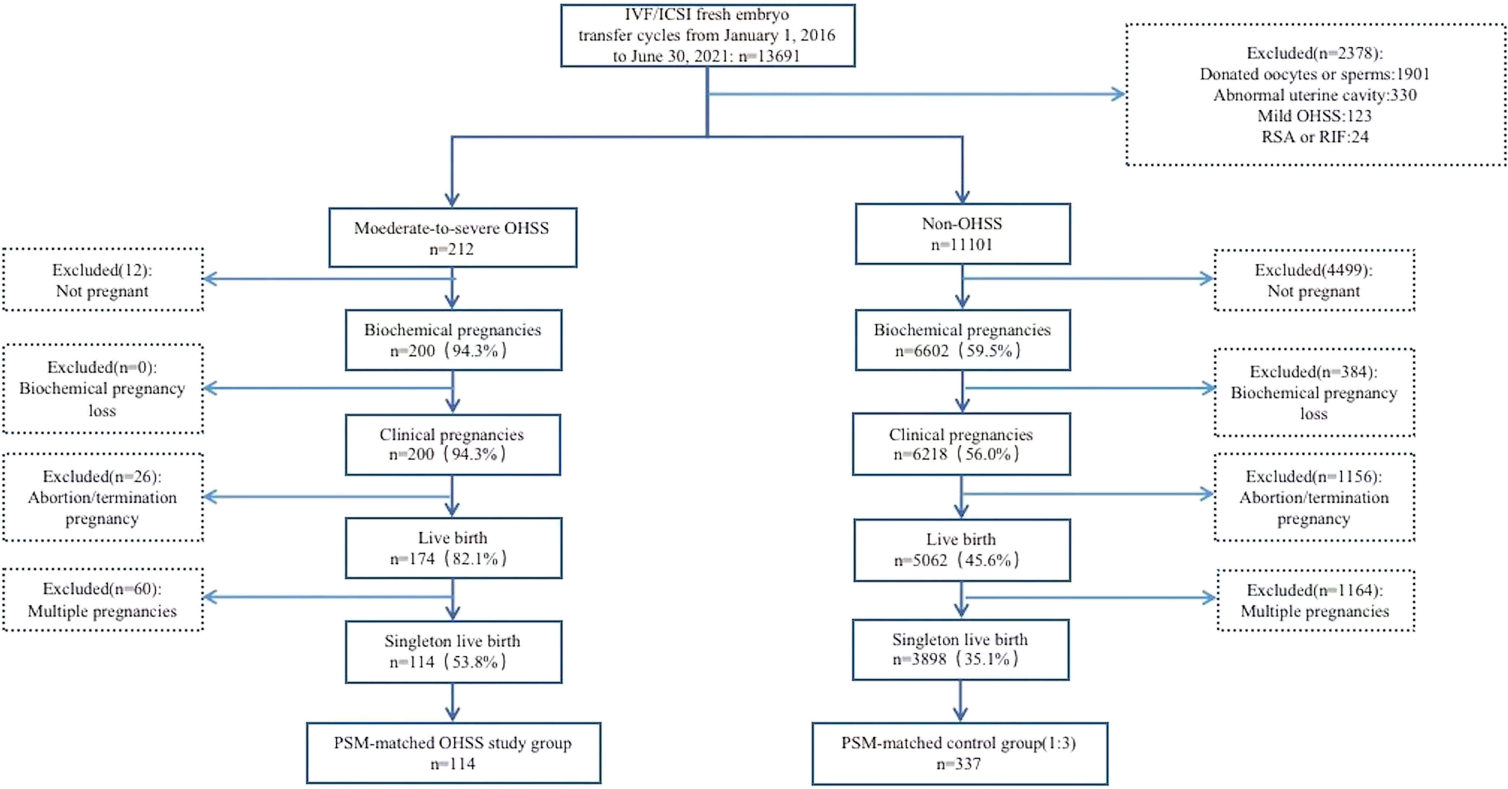
Flow diagram of study participants *IVF, in vitro fertilization; ICSI, intracytoplasmic sperm injection; OHSS, ovarian hyperstimulation syndrome; RSA, recurrent spontaneous abortion; RIF, repeated implantation failure; PSM, prospensity score matching*.

As shown in **Figure 1**, screening of the medical records revealed that 13,691 IVF-/ICSI-fresh ET cycles were performed from January 2016 to June 2021 in our center. Of these, 114 late MS-OHSS cycles and 3,898 non-OHSS cycles that met the inclusion criteria were included in this study. After PSM, 114 late MS-OHSS singleton live births and 337 non-OHSS singleton live births were included.

### Participant characteristics

**Table 1** shows that, compared with patients without OHSS, those with late MS-OHSS in our center were younger (*P* < 0.01) and had a lower body mass index (*P* = 0.02), lower basal serum follicle-stimulating hormone concentrations (*P* < 0.01), higher basal serum anti-Mullerian hormone concentrations (*P* < 0.01), and higher antral follicle counts (*P* < 0.01). Furthermore, there were significant differences between the late MS-OHSS and non-OHSS group in terms of the stimulation protocol (*P* = 0.04), day of ET (*P* < 0.01), number of embryos transferred (*P* < 0.01), and number of oocytes retrieved (*P* < 0.01). However, after PSM, all of the baseline characteristics were similar between the two groups.

**Table 1:** Baseline characteristics before and after propensity score matching.

	Before propensity score matching			After propensity score matching		
	MS-OHSS singleton pregnancies (n = 114)	Non-OHSS singleton pregnancies (n = 3,898)	*P* value	MS-OHSS singleton pregnancies (n = 114)	Non-OHSS singleton pregnancies(n = 337)	*P* value
Maternal age (y)	28.98 ± 3.56	30.45 ± 4.14	<0.01	28.98 ± 3.56	29.43 ± 3.76	0.27
Paternal age (y)	30.61 ± 5.50	31.38 ± 4.78	0.09	30.61 ± 5.50	30.49 ± 4.26	0.83
Maternal body mass index(kg/m2)	22.87 ± 2.79	23.60 ± 3.29	0.02	22.87 ± 2.79	22.88 ± 2.98	0.96
Duration of infertility (y)	3.10 ± 2.31	3.40 ± 2.63	0.22	3.10 ± 2.31	3.24 ± 2.27	0.68
Maternal gravidity (≥1)	55(48.25)	2,108(54.08)	0.22	55(48.25)	170(50.45)	0.56
Hormone level						
Basal serum FSH level (IU/L)	6.10 ± 1.90	6.84 ± 2.05	<0.01	6.10 ± 1.90	6.07 ± 1.69	0.88
Basal serum E2 level (pmol/L)	161.81 ± 131.16	155.82 ± 98.24	0.53	161.81 ± 131.16	155.94 ± 98.57	0.62
Basal serum LH level (IU/L)	6.86 ± 7.25	5.94 ± 5.36	0.18	6.86 ± 7.25	6.27 ± 5.88	0.38
Basal serum AMH level (pmol/L)	37.13 ± 19.95	28.33 ± 20.85	<0.01	37.13 ± 19.95	36.10 ± 27.68	0.67
Serum E2 level on the hCG trigger day (pmol/L)	10175.38 ± 7282.36	9182.46 ± 6463.62	0.11	10175.38 ± 7282.36	10833.96 ± 7090.96	0.40
Serum LH level on the hCG trigger day (IU/L)	1.48 ± 1.10	1.55 ± 1.84	0.70	1.48 ± 1.10	1.43 ± 0.90	0.60
Basal antral follicle count	20.68 ± 5.41	17.66 ± 6.95	<0.01	20.68 ± 5.41	20.63 ± 9.65	0.96
Stimulation protocol			0.04			0.69
Early follicular long-term protocol (%)	87(76.32)	2,637(67.65)		87(76.32)	242(71.81)	
Luteal medium long-term protocol (%)	12(10.53)	759(19.47)		12(10.53)	48(14.24)	
Antagonist protocol (%)	8(7.02)	353(9.06)		8(7.02)	29(8.61)	
Other protocol (%)	7(6.14)	149(3.82)		7(6.14)	18(5.34)	
Infertility diagnosisa			0.91			0.96
Male factor (%)	16(14.04)	599(15.37)		16(14.04)	50(14.84)	
Female factor (%)	62(54.39)	2,176(55.82)		62(54.39)	190(56.38)	
Couple factor (%)	27(23.68)	822(21.09)		27(23.68)	73(21.66)	
Unexplained (%)	9(7.89)	301(7.72)		9(7.89)	24(7.12)	
Treatment type			0.65			0.94
IVF (%)	87(76.32)	2,901(74.42)		87(76.32)	256(75.96)	
ICSI (%)	27(23.68)	997(25.58)		27(23.68)	81(24.04)	
Day of embryo transferred			<0.01			0.89
Day 3 (%)	54(47.37)	2,547(65.34)		54(47.37)	157(46.59)	
Day 5 or 6 (%)	60(52.63)	1,351(34.66)		60(52.63)	180(53.41)	
No. of embryo transferred			<0.01			0.87
1 (%)	66(57.89)	1,646(42.23)		66(57.89)	198(58.75)	
2 (%)	48(42.11)	2,252(57.77)		48(42.11)	139(41.25)	
No. of oocytes retrieved	16.11 ± 5.22	12.64 ± 5.3	<0.01	16.11 ± 5.22	16.18 ± 5.71	0.91
Endometrial thickness at the day of embryo transfer	11.81 ± 5.29	11.36 ± 2.22	0.37	11.81 ± 5.29	11.5 ± 2.13	0.55

Data are presented as mean ± standard deviation or n (%). The variables in the propensity score matching included maternal age, maternal body mass index, basal serum FSH level, basal serum AMH level, type of stimulation protocol, basal antral follicle count, number of oocytes retrieved, day of embryo development for embryo transfer, number of embryo transferred. MS-OHSS, moderate-to-severe ovarian hyperstimulation syndrome; FSH, follicle-stimulating hormone; LH, luteinizing hormone; E2, estradiol; AMH, anti-Müllerian hormone; hCG, human chorionic gonadotropin; IVF, *In vitro* fertilization; ICSI, intracytoplasmic sperm injection.

### Perinatal outcomes

The perinatal outcomes of the late MS-OHSS and non-OHSS group are presented in **Table 2**. After univariate and multivariate regression, the rates of normal term infant, placenta previa, PA, PROM, HDP, GDM, cesarean delivery, preterm birth, post-term birth, macrosomia, LBW, LGA, admission to the pediatrics department, and birth defect were not significantly different between the late MS-OHSS and non-OHSS groups. In addition, the other perinatal outcomes examined in this study (gestational age, birth weight, birth height, and 1-minute Apgar scores) did not differ significantly between the late MS-OHSS and non-OHSS groups. After PSM, as shown in **Table 3**, the risk of perinatal outcomes was not significantly different between the late MS-OHSS and matched non-OHSS groups.

**Table 2:** Perinatal outcomes among MS-OHSS patients and Non-OHSS patients before propensity score matching.

	MS-OHSS singleton pregnancies (n = 114)	Non-OHSS singleton pregnancies (n = 3,898)	Odds ratio/mean difference(95% CI)	Adjusted odds ratio/mean difference (95% CI)
Obstetric outcomes				
Placenta previa (%)	0(0.00)	38(0.97)	-	-
Placenta abruption (%)	0(0.00)	17(0.44)	-	-
Premature rupture of membranes (%)	4(3.51)	140(3.59)	0.98(0.35-2.68)	1.04(0.37-2.89)
Hypertensive disorders in pregnancy (%)	4(3.51)	156(4.00)	0.87(0.32-2.40)	1.07(0.39-2.98)
Gestational diabetes mellitus (%)	5(4.39)	356(9.13)	0.46(0.19-1.13)	0.57(0.23-1.41)
Cesarean delivery (%)	73(64.04)	2,508(64.34)	0.91(0.62-1.34)	1.10(0.74-1.64)
Neonatal outcomes				
Normal term infant (%)	98(85.96)	3,232(82.91	1.26(0.74-2.16)	1.15(0.67, 1.98)
Gestational age (week)	39.17 ± 1.30	39.03 ± 1.63	0.13(-0.17-0.44)	0.05(-0.26-0.35)
Premature delivery (%)	6(5.26)	273(7.00)	0.74(0.32-1.69)	0.83(0.36-1.92)
Birth weight (g)	3315.35 ± 439.30	3368.90 ± 521.04	-53.55(-150.22-43.11)	-47.37(-143.81-49.08)
Low birth weight (%)	4(3.51)	154(3.95)	0.88(0.32-2.43)	0.98(0.35-2.70)
Very low birth weight (%)	0(0.00)	25(0.64)	-	-
Macrosomia (%)	6(5.26)	318(8.16)	0.63(0.27-1.43)	0.67(0.29-1.54)
SGA (%)	4(3.51)	75(1.92)	1.85(0.67-5.16)	1.76(0.62-4.98)
LGA (%)	26(22.81)	1,101(28.25)	0.75(0.48-1.17)	0.85(0.54-1.33)
Admitted in pediatrics (%)	1(0.88)	70(1.80)	0.48(0.07-3.51)	0.58(0.08-4.26)
Birth defect (%)	1(0.88)	46(1.18)	0.74(0.10-5.42)	0.88(0.12-6.57)
Birth height (cm)	50.22 ± 1.49	50.24 ± 2.06	-0.02(-0.40-0.36)	-0.01(-0.39-0.38)
1-minute Apgar scores	10.00 ± 0.00	9.96 ± 0.38	0.04(-0.03-0.11)	0.05(-0.02-0.12)

Data are presented as mean ± standard deviation or n (%). logistic regression and linear regression were used to calculate odds ratio (95% CI) and mean difference (95% CI). Adjusted odds ratio (95% CI) and mean difference (95% CI) adjusted for maternal age, maternal body mass index, basal serum FSH level, basal serum AMH level, type of stimulation protocol, basal antral follicle count, number of oocytes retrieved, day of embryo development for embryo transfer, number of embryo transferred. MS-OHSS, moderate-to-severe-ovarian hyperstimulation syndrome; CI, confidence interval; -, not applicable; SGA, small for gestational age; LGA, large for gestational age.

**Table 3:** Perinatal outcomes among MS-OHSS patients and Non-OHSS patients after propensity score matching.

	MS-OHSS singleton pregnancies (n = 114)	Non-OHSS singleton pregnancies (n = 337)	Odds ration/mean difference (95% CI)	*P* value
Obstetric outcomes				
Placenta previa (%)	0(0.00)	3(0.89)	-	-
Placenta abruption (%)	0(0.00)	2(0.59)	-	-
Premature rupture of membranes (%)	4(3.51)	8(2.37)	1.50(0.44,5.06)	0.52
Hypertensive disorders in pregnancy (%)	4(3.51)	9(2.67)	1.33(0.40,4.39)	0.64
Gestational diabetes mellitus (%)	5(4.39)	26(7.72)	0.55(0.21,1.46)	0.23
Cesarean delivery (%)	73(64.04)	208(61.72)	1.10(0.71,1.72)	0.66
Neonatal outcomes				
Normal term infant (%)	98(85.96)	287(85.16)	1.07(0.58, 1.96)	0.83
Gestational age (week)	39.17 ± 1.30	39.20 ± 1.34	-0.04(-0.32, 0.25)	0.80
Premature delivery (%)	6(5.26)	14(4.15)	1.28(0.48,3.42)	0.62
Birth weight (g)	3315.35 ± 439.30	3372.16 ± 478.35	-56.81(-156.64, 43.02)	0.26
Low birth weight (%)	4(3.51)	13(3.86)	0.91(0.29,2.84)	0.87
Very low birth weight (%)	0(0.00)	1(0.30)	-	-
Macrosomia (%)	6(5.26)	26(7.72)	0.66(0.27,1.66)	0.38
SGA (%)	4(3.51)	8(2.37)	1.50(0.44,5.06)	0.52
LGA (%)	26(22.81)	92(27.30)	0.79(0.48,1.30)	0.35
Admitted in pediatrics (%)	1(0.88)	3(0.89)	0.99(0.10,9.57)	0.99
Birth defect (%)	1(0.88)	3(0.89)	0.99(0.10,9.57)	0.99
Birth length (cm)	50.22 ± 1.49	50.31 ± 1.61	-0.09(-0.43, 0.24)	0.59
1-minute Apgar scores	10.00 ± 0.00	9.99 ± 0.13	0.01(-0.01, 0.04)	0.34

Data are presented as mean ± standard deviation or n (%). Univariate logistic regression and linear regression were used to calculate odds ratio (95% CI) and mean difference (95% CI). MS-OHSS, moderate-to-severe ovarian hyperstimulation syndrome; CI, confidence interval; -, Not applicable; SGA, small for gestational age; LGA, large for gestational age.

## Discussion

The results of our data showed that the perinatal outcomes were similar between MS-OHSS group and non-OHSS group. The perinatal outcomes of pregnancies complicated by MS-OHSS have not yet been thoroughly investigated. Our results were similar to several previous reports (15, 16, 30). The study by Choux et al. (16) included 77 patients hospitalized for severe OHSS and 231 matched patients without OHSS, only 55 OHSS patients achieved singleton live births. They found the perinatal outcomes were similar between the OHSS group and non-OHSS group. A retrospective cohort study of 190 patients with OHSS in China showed that OHSS did not exert any obviously adverse effect on subsequent pregnancies (15). The authors further analyzed the differences in the rates of multiple pregnancy. But they did not separately compare the perinatal outcomes of patients who achieved singleton live births. Jia et al. (30) also demonstrated similar outcomes to our study. However, they only included patients with mild and moderate OHSS and did not assess the effects of OHSS on obstetric complications. As patients with mild OHSS only have minor clinical symptoms and the disorder is self-limiting (6, 31), few patients with mild OHSS require admission to hospital for treatment. Some patients may only experience mild bloating and are unaware of having OHSS. Therefore, it is challenging to estimate the actual sample size of patients with mild OHSS. Thus, only patients with MS-OHSS were included in our study. Hu et al. (22) used a study design similar to ours but obtained conflicting results. They used PSM to match the rate of multiple gestations rather than analyzed the obstetric and neonatal outcomes in OHSS patients who achieved single live births separately. They found that late MS-OHSS increased the risk of GDM, thrombosis, and NICU hospitalization. Schirmer et al. (21) analyzed National Assisted Reproductive Technology Surveillance System data and found that patients with OHSS had higher risks of preterm delivery and LBW than those without OHSS.

OHSS is a self-limiting disorder associated with hormones (6, 9). The occurrence of OHSS is correlated with administration of HCG and concentrations of estradiol (E_2_) (16, 32). HCG promotes the fusion of cytotrophoblasts to create syncytiotrophoblasts and helps to remodel spiral arteries, thus improving the propensity of implantation and playing a role in the success of pregnancies in the presence of OHSS (16). Depending on the time of occurrence, OHSS is classified as early OHSS (occurring 9 or fewer days after oocyte retrieval) or late OHSS (occurring no sooner than 10 days after oocyte retrieval) (33). However, the pathogeneses of these two types of OHSS are distinct. Early OHSS is an acute response of multiple follicles to exogenous HCG given in a short period of time, while late OHSS is usually caused by endogenous HCG secreted by trophoblast cells after achieving pregnancy (8, 33, 34). Compared with early OHSS, the stimulation caused by endogenous HCG in late OHSS was slower (33). There may be adaptive processes in the organism. This hypothesis need to be confirmed in future studies. The MS-OHSS patients included in our study were all late onset. Furthermore, OHSS with abnormal transient hemodynamics usually occurs only in the first trimester of pregnancy. This implies that OHSS may have less impact on subsequent pregnancies.

It was proposed that serum concentrations of E_2_ are a predictor of OHSS in women undergoing ART (32). Some studies have shown that supraphysiological concentrations of E_2_ after controlled ovarian hyperstimulation negatively affect embryo quality, extravillous trophoblast invasion, endometrial receptivity, and placental development (35–37). Moreover, studies by researchers at our center have demonstrated that supraphysiological E_2_ concentrations (≥ 4,000 pg/ml) on the day of HCG injection increase the risks of singleton SGA, LBW, and full-term LBW (38). However, in our study, the serum E_2_ concentrations on the day of HCG injection or the basal serum E_2_ concentrations were similar between the MS-OHSS and non-OHSS groups. This may be related to the treatment strategy adopted for these patients. To prevent severe overstimulation, we recommend patients whose ovaries are enlarged before transplantation or whose serum E_2_ concentrations are excessively high on the day of HCG injection to cancel the transfer and freeze all the embryos. Transferring the frozen embryos later may guarantee an acceptable reproductive outcome. This may also be the reason why there was no difference in perinatal outcomes between MS-OHSS group and non-OHSS group.

### Strengths and limitations

We analyzed the effects of late MS-OHSS on the perinatal outcomes of patients who achieved single live births after IVF-/ICSI-fresh ET cycles. Moreover, the influence of various confounding factors on the perinatal outcomes were controlled by PSM and regression. Therefore, our study results provide useful insights for both clinicians and patients. However, our study has some limitations. First, the sample size of patients with MS-OHSS was limited. Therefore, our observations should be interpreted with caution, and a larger sample size will be used in our future study. Second, we did not compare the perinatal outcomes of patients with different severity levels of OHSS. Third, we did not analyze the hospitalization information of patients, such as the length of hospitalization and specific treatment received. Last, this study was a retrospective study. Therefore, the possibility of potential recall bias cannot be ignored.

## Conclusion

The perinatal outcomes of singleton live births in patients with MS-OHSS were similar to those in patients without OHSS. To confirm this conclusion, we will adopt a larger sample size in a future study. In addition, further studies are warranted to evaluate the impact of OHSS on long-term prognosis by conducting long-term follow-up of patients and neonates, and to further explore the pathogenesis of OHSS through basic experiments.

## Data availability statement

The original contributions presented in the study are included in the article/supplementary material. Further inquiries can be directed to the corresponding author.

## Ethics statement

This retrospective cohort study was approved by the ethics committee of the Third Affiliated Hospital of Zhengzhou University (ethics approval number: 2022-164-01).

## Author contributions

YG and SR designed the study, selected the population to be included and excluded, extracted and analyzed data and drafted of the manuscript. RZ, HW, BR, WenZ and JD were involved in the data collection and statistical analysis. WZ, CY, SY, and JD contributed to the revising of the manuscript. All authors contributed to the article and approved the submitted version.

## Funding

This research was supported by State Key Laboratory of Reproductive Medicine, Nanjing Medical University (SKLRM-K201903), Henan young and middle-aged health science and technology innovation leading talent training project (YXKC2021020) and National Health Commission Scientific Research Foundation Henan Medical Science and Technology Research Program Provincial Joint Construction Project (SBGJ202102180).

## Acknowledgments

The authors acknowledge all the patients participated in the study and the participants contributed to this study.

## Conflict of interest

The authors declare that the research was conducted in the absence of any commercial or financial relationships that could be construed as a potential conflict of interest.

## Publisher’s note

All claims expressed in this article are solely those of the authors and do not necessarily represent those of their affiliated organizations, or those of the publisher, the editors and the reviewers. Any product that may be evaluated in this article, or claim that may be made by its manufacturer, is not guaranteed or endorsed by the publisher.
